# Gender Disparities in Patients' Decisions about the Management of Myocardial Infarction in East Chinese Province

**DOI:** 10.1155/2023/8220308

**Published:** 2023-12-06

**Authors:** Chaobin Lou, Tao Xu, Fangying Dong, Yangmiao Xu, Muhua Zhang, Shudong Xia, Yinchuan Xu, Chao Feng

**Affiliations:** ^1^Department of Cardiology, The Fourth Affiliated Hospital of Zhejiang University, Yiwu 322000, Zhejiang, China; ^2^Department of Emergency Medicine, The Second Hospital of Jiaxing, The Second Affiliated Hospital of Jiaxing University, Jiaxing 314000, Zhejiang, China; ^3^Department of Cardiology, The Second Affiliated Hospital of Zhejiang University, Hangzhou 310000, Zhejiang, China

## Abstract

**Background:**

Previous studies showed that there are gender disparities in various respects of acute myocardial infarction (AMI), including risk factors, symptoms, and outcomes. However, few of them noticed the gender disparities in patients' decision about the management of AMI, which might also be associated with the outcome.

**Aims:**

To identify gender disparities in patients' decisions about the management of myocardial infarction.

**Methods:**

In this cohort study, the critical time points including the time of symptom onset, visiting hospital, diagnosis of AMI, consent to coronary angiography (CAG), beginning of CAG, and balloon dilation were recorded. Medication and major adverse cardiac event (MACE) within 6 months were also recorded.

**Results:**

Female patients took more time from symptom onset to visiting hospital (*P* = 0.001), from diagnosis of AMI to consent to CAG (*P* < 0.05), and from door to needle/balloon than male (*P* < 0.05). Less female patients accepted CAG (*P* < 0.05) and coronary intervention/bypass grafting (*P* < 0.05). Less female patients kept good inherence to antiplatelet therapy (*P* < 0.05) and statins (*P* < 0.05) than male, more female preferred traditional Chinese medicine (TCM) than male patient (*P* < 0.05), and most of them had MACE within 6 months (*P* < 0.05). Patients' good adherence to antiplatelet therapy and statins and accepting coronary intervention/bypass grafting were associated with a reduced risk of MACE.

**Conclusion:**

Female patients were more reluctant to make decisions about emergency management of AMI and tended to choose conservative treatment. More female patients preferred TCM than evidence-based medicine. Their reluctance about the critical management of AMI and poor adherence to evidence-based medicine were associated with an elevated risk of MACE.

## 1. Introduction

Acute myocardial infarction (AMI) is one of the leading causes of death worldwide for both men and women [1, 2]. For decades, plenty of studies focused on the epidemiology, pathogenesis, and treatment of AMI with accumulating evidence showing gender differences in various aspects including risk factors and outcomes. Compared with men, women had lower prevalence of AMI, higher adverse events after treatment, and higher in-hospital and long-term mortality [3–6].

Based on vast evidence, various guidelines have supplied detailed recommendations for the management of AMI [7–9]. Experienced doctors implement the guidelines and make recommendations to patients from prevention to intervention. In China, cardiologists have been trying to follow the guidelines for decades, including the use of coronary angiography (CAG) and percutaneous coronary intervention (PCI) for patients with severe coronary stenosis, dual antiplatelet therapy (DAPT), and statins for secondary prevention after AMI, and have already made great success in reducing the mortality after AMI [10–12]. However, the appropriate application of evidence-based medicine and guideline-recommended therapy still requires improvement. For example, some patients of AMI would not accept CAG or PCI, while traditional Chinese medicine (TCM) which is criticized for lack of reliable clinical evidence is still the first choice for a large number of patients [13–15]. Therefore, the management of AMI is realistically based on the combination of doctors' recommendations and patients' decisions [16, 17]. It is the patient who decides when to call ambulance or go to hospital after chest pain, whether to accept doctors' recommendation about CAG or PCI, whether to keep long-term medication after discharge, and whether to choose traditional Chinese medicine (TCM) as the main medication or supplement. Patients of different genders might have different decisions which lead to different outcomes.

Previously, most studies focused on the characteristics of AMI including risk factors, manifestations, radiological findings, and treatment responses [18, 19]. However, few studies noticed the gender disparities in patients' decisions about the emergency and long-term management of AMI. In this study, based on the data of a regional chest center where every critical procedure and every time point about patients' critical decisions were recorded, we aimed to investigate the gender disparity in patients' decisions about the management of AMI and the impact on the prognosis of AMI.

## 2. Materials and Methods

### 2.1. Study Population and In-Hospital Management

This was a cohort study in the Fourth Affiliated Hospital of Zhejiang University. From May 1, 2016, to May 1, 2021, all patients were registered and screened if they attended to the Chest Pain Center of the Fourth Affiliated Hospital of Zhejiang University. This hospital is located in Yiwu City, covers 1 million residents, and is less than one hour's drive from most communities of Yiwu in distance. Patients were enrolled if they were diagnosed as AMI for the first time with consent to this study. The diagnosis and management of AMI was based on the recommendation of guidelines from ESC. Specifically, in the Chest Pain Center, an electrocardiogram was performed immediately for every patient reporting acute chest pain. Blood tests were performed to identify the level of cardiac troponin, creatine kinase, creatine kinase-MB, myoglobin, blood cell counts, serum creatinine, electrolytes, coagulation function, and D-dimer immediately. Patients were diagnosed to have ST-elevated myocardial infarction (STEMI) based on the results of electrocardiogram or non-ST-elevated myocardial infarction (NSTEMI) based on the results of electrocardiogram, troponin, and symptoms including chest pain, chest distress, back pain, abdominal pain, and syncope. A package of medication including aspirin, clopidogrel/ticagrelor, and statins were prescribed immediately for every patient with AMI and without contraindications of medication. CAG was recommended for every patient suspected to have AMI without obvious contradictions, with the benefits and potential complications of CAG also informed. PCI or coronary artery bypass grafting (CABG) was recommended for every patient with indications as shown by CAG. The strategy of PCI was based on the guideline and patients' decision, mainly including the culprit-lesion-only PCI (stent implantation or drug-eluted balloon dilation) for most patients and complete revascularization for some patients. The critical time points including the time when patients had chest pain, when patients arrived at the hospital, when the diagnosis of AMI was made, when the patients agreed to have CAG, when the CAG began, and when the first balloon was dilated were recorded. The time between symptom onset to visiting hospital, time between diagnosis of AMI and consent to CAG, time of door-to-needle for NSTEMI, and time of door-to-balloon for STEMI were calculated, respectively, for each patient.

During hospitalization, information including height and weight, gender and age, education level (education no less than 6 years were deemed as basic education), marriage status, living address, valid phone number, previous medical history, and comorbidities including hypertension, diabetes, hyperlipidemia, stroke, chronic kidney disease, and peripheral artery disease were obtained. Patient with body weight index more than 28 was identified to be obese [20–22]. Guideline-recommended medications including DAPT, statins, beta-blockers, and angiotensin-converting enzyme inhibitors (ACEIs)/angiotensin receptor blockers (ARBs) were prescribed for every patient without contradictions. Anticoagulation was initiated for patients with atrial fibrillation/flutter, cardiac thrombosis, or deep vein thrombosis. Education about the necessity of long-term medication, diet control, smoking cessation, alcohol control, and physical exercise was performed during hospitalization and before discharge. At discharge, abovementioned oral medications were prescribed with education performed again by one certificated doctor and nurse with the context listed in a typed discharge form for every patient.

Patients in requirement of cardiac resuscitation in the chest pain center, patients who died during hospitalization, patients with AMI caused by conditions other than primary coronary artery disease (AMI of type II, III, IV, and V) [7], patients in the end-stage of chronic diseases with life expectancy less than half a year, patients with severe cognitive dysfunction, hemiplegia, or other severe mental or brain diseases that impaired their ability of drug-taking willingly, and patients with severe hematological diseases and liver diseases that enabled the use of antiplatelet and statins were excluded.

The primary endpoint of this study was any one of the major adverse cardiovascular events (MACEs) within 6 months after discharge. The presence of MACE was defined as death of any reason, congestive heart failure which required hospitalization, i.e., intravenous diuretics or vasoactive agents, recurrence of myocardial infarction, and stroke of any subtype. For every patient visiting hospital with MACE, a face-to-face interview was performed about their medication in the last week and reasons why they discontinued the medications including DAPT and statins. During the interview, patients (or their family members for several patients) were asked about their current medication, and “Have you ever missed the aspirin/clopidogrel/ticagrelor/statins prescribed by your doctor deliberately or inadvertently?” As introduced in our previous study [23], patients' adherence to medications was evaluated using an adherence score (0–4), which was assigned to each patient according to their answer combined with the records of hospital/pharmacy-visiting and medicine-purchasing, with the definition of each score listed in Table 1. Adherence to antiplatelet therapy and statins was recorded, respectively.

During hospitalization, TCM was not used. Patients would not be asked about their tendency of taking TCM. After discharge, although patients were encouraged to get all the medications in the outpatient department during follow-up, some of them tended to get them in qualified pharmacies or TCM clinics, where TCM might be chosen by them as main treatment or supplement. TCM was usually manufactured into ready-made medicine (tablets or capsule, several tablets/capsules twice or three times a day according to instruction). The use of TCM and the frequency were also recorded in the interview. Using TCM for at least twice a week was regarded as regular use of TCM. For patients without MACE, after 6 months, the abovementioned interview and questionnaire were also performed face-to-face in the outpatient clinic to identify their adherence to antiplatelet drugs, statins, and the use of TCM.

### 2.2. Statistical Analysis

All data analysis was performed using SPSS 18.0. Categorical data were listed as percentage (number). Ordinal categorical data and continuous data were listed as mean ± standard deviation. Characteristics were compared between female and male patients with AMI, and also between female and male patients in STEMI and NSTEMI subgroups, respectively. Specifically, categorical data including the presence of various vascular risk factors and comorbidities, social-economic factors, percentage of accepting CAG and PCI/CABG, regular use of TCM, and MACE were compared using chi-square test between female and male patients. Ordinal categorical data including the adherence to antiplatelet drugs and statins were compared using Kruskal–Wallis test. Continuous data including age, time between symptom onset to visiting hospital, time between diagnosis to consent to CAG, and time of door-to-needle and door-to-balloon were compared using students' *T* test. Logistical regression was performed to identify the independent risk factor for MACE of patients of STEMI and NSTEMI, respectively, with age, gender, all the vascular risk factors, comorbidities, social-economic factors, acceptance of PCI/CABG, scores of adherences to antiplatelet drugs and statins, and regular use of TCM added in the models. *P* ≤ 0.05 was identified to indicate statistical significance.

The sample size estimation of this study was based on the “events per variable” (EPV) method which is a classical and easy method for the estimation of sample size without calculation using special software. Specifically, EPV was presumed to be 10, the rate of endpoint events (MACE) was estimated to be 15%, and the number of covariates were 15; therefore, the estimated sample size should be approximately 1000. Patients were consecutively enrolled and sampled into different groups according to their diagnosis.

## 3. Results

**Figure 1:**
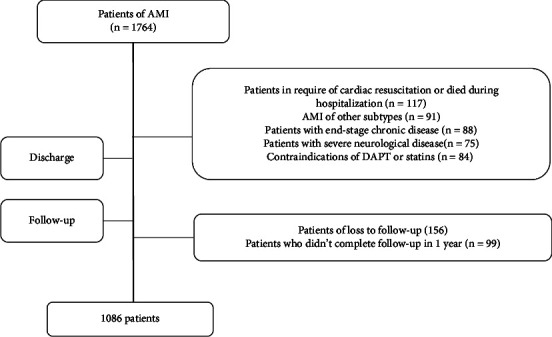
Flowchart of study enrollment.

From January 1, 2016, to May 1, 2021, 1764 patients were screened and diagnosed to have AMI. 678 patients were excluded due to various reasons in the exclusion criteria (*n* = 423), loss to follow-up (*n* = 156), or quitted follow-up (*n* = 99). 1086 patients were enrolled after follow-up (details in Figure 1). Among all the patients enrolled in this study, 472 patients were female, 614 patients were male, 654 patients had NSTEMI, and 432 patients had STEMI.

The basic characteristics of all patients are listed in Table 2. The comparison between female and male patients of AMI showed that female patients were older (69.0 y vs 63.0 y), had lower level of education, higher prevalence of hypertension (71.8% vs 63.2%), diabetes (41.6% vs 31.4%), hyperlipidemia (83.5% vs 77.4%), and significant lower prevalence of smoking (9.5% vs 40.7%) and drinking (19.1% vs 44.1%). Besides, more male patients were prescribed with the combination of aspirin and ticagrelor than female patients (74.8% vs 60.2%) and more male patients lived with their spouses (84.4% vs 78.2%). The comparison between female and male patients in STEMI and NSTEMI subgroups showed similar results as mentioned above, except for the education level in STEMI subgroup and prevalence of hyperlipidemia in NSTEMI subgroup were not statistically different between female and male patients.

The comparison of patients' critical decisions about the management of AMI showed that, in NSTEMI subgroup, female patients took more time from symptom onset to visiting hospital, from the diagnosis of AMI to make consent to CAG, and from visiting hospital to CAG (door-to-needle) than male patients. In STEMI subgroup, female patients took more time from diagnosis of AMI to make consent to CAG with a longer time of door-to-balloon than male patients. In both STEMI and NSTEMI subgroups, less female patients agreed to CAG and PCI/CABG, less female patients strictly keep adherence to antiplatelet drugs and statins, more female patients took TCM. In STEMI subgroup, 48 patients (11.1%) had MACE, including 2 deaths, 29 heart failure, 16 recurrent AMI, and 3 stroke. In NSTEMI subgroup, 75 patients (11.5%) had MACE, including 4 deaths, 40 heart failure, 35 recurrent AMI, and 5 stroke. The comparison showed that in both subgroups, female patients has higher prevalence of MACE; however, only in the STEMI subgroup, the result of the comparison had statistical significance (*P* < 0.05). Details about patients' decisions and MACE are listed in Tables 3 and 4.

The multivariate analysis for the risk factors of MACE showed that, decision to accept PCI/CABG and good adherence to DAPT were independently associated with a lower risk of MACE for both patients with STEMI and NSTEMI, good adherence to statins was independently associated with a lower risk of MACE for patients with STEMI, while regular use of TCM was not associated with MACE for both STEMI and NSTEMI groups. Details about the results of multivariate analysis are listed in Table 5.

## 4. Discussion

This study showed that there were distinct gender disparities in patients' decisions about the management of AMI. Compared with male patients, female patients were associated with more delay in critical decisions including going to hospital, accepting CAG after diagnosis of AMI and further PCI or CABG, thus had higher risk of MACE after AMI than male. Besides, more female patients kept poor adherence to antiplatelet therapy and statins. Their poor adherence to evidence-based medicine was also independently associated with MACE after AMI especially after STEMI. Instead, more female patients preferred TCM than male patients. However, regular use of TCM seemed helpless in reducing the risk of MACE after AMI. As far as we know, this was the first report about the gender disparities in patients' decisions about the emergency and long-term management of AMI.

This study showed obvious gender disparities in various aspects of AMI including vascular risk factors, socioeconomic factors, and outcomes. Besides, gender disparities seemed to be consistent in both STEMI and NSTEMI subgroups. In risk factors, female patients of AMI were much older, had higher prevalence of traditional vascular risk factors including hypertension, diabetes, and hyperlipidemia, while few of them were smokers or alcoholic. The gender disparities in vascular risk factors were in consistent with previous reports [6, 24] and were not the key point of this study.

One of the major findings was the obvious gender disparities in patients' decisions about the emergency management of AMI. Actually, previous studies had already noticed that women were more reluctant to seek for help than men after chest pain, partly because their chest pain might be less severe and less typical [24, 25]. This study showed that female patients were reluctant in nearly every key step about the emergency management of AMI, not just in seeking medical help. Female patients were less likely to accept CAG and took longer time to make consent to CAG. For patients who underwent CAG, female patients were less likely to accept PCI or CABG and, therefore, were more likely to miss the best time to save the myocardium. Previous report showed that the less women of AMI accepted timely PCI or primary PCI within 90 minutes in China [6]. Based on the results of this study, the delay of PCI for women could be attributed to their reluctance to accept CAG to some extent. The reason for their reluctancy might be beyond the biological factors such as the atypical chest pain which might not be that severe and is more prevalent among women [24]. Social-economic factors might also be the reasons for the gender disparities in critical decisions. Female patients of AMI were much older than men, less of then completed basic education and lived with their spouses who were the major sources of help and consultations for every patient. It is reasonable that female patients take longer time to understand the severity of AMI, the necessity of CAG and PCI based on a poor education level and less social support.

The management of AMI is not just based on the emergency medication and PCI but also requires long-term medication especially antiplatelet therapy and statins [7, 8]. This study showed that poor adherence to antiplatelet therapy and statins was independent risk factors for MACE after AMI. Compared with male patients, female patients were more likely to have poor adherence to the abovementioned medications. This might also be one of the major causes for the higher prevalence of MACE among female patients after AMI. The adherence to long-term medication has always been a big challenge for the management of chronic diseases including heart disease [26, 27]. Although patients with chronic diseases would be informed about the necessity and benefit of long-term medication, 50% patients would not take their medications as advised according to a report published by WHO [28]. The reasons for the poor adherence to medications were complicated involving patients, doctors, and health systems [27, 29]. Lack of understanding of their disease and lack of social support were both patient-related factors associated with the poor adherence to medication. As shown in this study, female patients of AMI were much older, had lower levels of education with more of them living without their spouses, and therefore were more likely to neglect the necessity of long-term medication including antiplatelet therapy and statins. Besides, female patients of AMI were more likely to have complications caused by antiplatelet therapy and statins [30–32]. This might also be one of the reasons why more female patients of AMI were poorly adherent to antiplatelet therapy and statins.

Actually, female gender has been established as independent risk factor of adverse outcome after AMI [33, 34]. In this study, the results also showed that female patients of AMI had higher proportion of MACE. However, the results of logistic regression showed no statistical association between gender and MACE. It is possible that the strong association between gender and MACE is partly contributed to their decision about the treatment, such as acceptance of PCI/CABG and good adherence to long-term medication, which were demonstrated to be independent risk factors for MACE but were rarely studied in previous studies.

Another major finding of this study was about the use of TCM. This study showed that less women kept good adherence to antiplatelet medication and statins, while more of them would take TCM as major or supplement medications. However, regular use of TCM seemed not be able to reduce MACE. In China, modern medicine and TCM which are quite different in both theory and practice have been coexisting for decades. Sometimes they work together, sometimes they are competitors. In our previous study, the results even showed that the use of TCM was a major barrier for patients' adherent to antiplatelet therapy [23]. There are plenty of TCM that could be used for the management of AMI, mainly including TCM that could “promote blood flow, alleviate blood stagnation, and reduce blood viscosity” as claimed by pharmacies or pharmaceutic companies, such as the products of gingko, Asian ginseng, danshen, safflower, sanqi, and aflatoxin [35, 36], which were usually not cheaper than guideline-recommended medications. Although they were criticized for their flawed clinical trials or lack of clinical evidence [13, 15, 37] and were not recommended by main guidelines [7, 8], they were still entrusted by a lot of patients of AMI in China and were especially entrusted by female patients according to the results of this study. It is quite reasonable because most patients do not read or understand guidelines. On the contrary, they are more familiar with TCM because the names of TCM are usually easier to understand for Chinese, their medicine instructions are more attractive with magic effects listed and obscure side effects mentioned. Furthermore, many TCM-related products are permitted to be advertised on TV and sold in pharmacies as over-the-counter drugs. With the help of traditional media and pharmacies, together with the attachment to tradition, the use of TCM was further promoted.

The strength of this study mainly included the detailed analysis of patients' decisions and the main time points when patients made critical decisions based on the records of chest pain center. However, some limitations were inevitable. First, this study was based on a single center with limited sample size; therefore, the significance of the results was limited. Second, the time point of symptom onset was supplied by patients and was not accurate enough especially for those without severe chest pain. Third, there are still some factors beyond our investigation, such as the financial situation of the patients. In the future, more studies about the gender disparities of AMI are still required.

## 5. Conclusion

This study showed obvious gender disparities in critical decisions on the emergency and long-term management of AMI and proved their association with MACE. It suggested that we should make more effort in education about the symptom recognition, process of emergency management, the necessity of CAG, advantage of PCI, and the necessity of long-term medication especially for female patients of AMI to improve their outcome. At the social level, it is quite important to improve the social support for women and also improve the education level of medical science especially evidence-based medicine.

## Figures and Tables

**Table 1:** Evaluation of patients' adherence to long-term medication including DAPT and statins.

Adherence score	Definition
4	Patient claimed that he/she never missed any drug with full records of medicine-purchasing or just inadvertently missed the antiplatelet drugs or statins prescribed by the doctor occasionally
3	Patient missed either one of the DAPT or statins once or twice a week
2	Patient missed either one of the DAPT or statins more than twice a week
1	Patient admitted that he/she took either one of the DAPT or statins less than twice a week
0	Patient admitted that he already stopped antiplatelet therapy or statins for at least one week

DAPT: dual antiplatelet therapy.

**Table 2:** Gender disparities in the baseline characteristics of patients with AMI.

	All AMI patients			NSTEMI subgroup			STEMI subgroup		
	Female (*n* = 472)	Male (*n* = 614)	*P*	Female (*n* = 289)	Male (*n* = 365)	*P*	Female (*n* = 183)	Male (*n* = 249)	*P*
Age (y)	69.0 ± 10.3	63.0 ± 12.8	<0.001	69.5 ± 10.1	63.9 ± 12.3	<0.001	68.4 ± 10.6	61.6 ± 13.3	<0.001
Education ≥6 years, % (*n*)	47.9 (226)	55.2 (339)	0.010	47.8 (138)	56.4 (206)	0.017	48.1 (88)	53.4 (133)	0.285
Rural residency, % (*n*)	64.4 (304)	63.0 (387)	0.656	63.7 (184)	63.6 (232)	0.999	65.6 (120)	62.2 (155)	0.544
Living with spouse, % (*n*)	78.2 (369)	84.4 (518)	0.006	77.9 (225)	85.2 (311)	0.010	78.7 (144)	83.1 (207)	0.263
Hypertension, % (*n*)	71.8 (339)	63.2 (388)	0.002	73.4 (212)	64.7 (236)	0.011	69.4 (127)	61.0 (152)	0.084
Diabetes, % (*n*)	42.6 (201)	31.4 (183)	<0.001	40.5 (117)	31.5 (115)	0.011	45.9 (84)	31.3 (78)	0.001
Hyperlipidemia, % (*n*)	83.5 (394)	77.4 (475)	0.007	83.4 (241)	80.5 (294)	0.360	83.6 (153)	72.7 (181)	0.005
Stroke, % (*n*)	10.8 (51)	12.5 (77)	0.394	10.4 (30)	12.9 (47)	0.393	11.5 (21)	12.0 (30)	0.881
Peripheral vascular disease, % (*n*)	16.7 (79)	13.4 (82)	0.122	15.2 (44)	13.7 (50)	0.654	19.1 (35)	12.9 (32)	0.081
Obesity, % (*n*)	27.5 (130)	23.6 (145)	0.159	25.6 (74)	23.0 (84)	0.463	30.6 (56)	24.5 (61)	0.189
Smoking, % (*n*)	9.5 (45)	40.7 (250)	<0.001	9.0 (26)	40.3 (147)	<0.001	10.4 (19)	41.4 (103)	<0.001
Drinking, % (*n*)	19.1 (90)	44.1 (271)	<0.001	19.7 (57)	46.3 (169)	<0.001	18.0 (33)	41.0 (102)	<0.001
DAPT subtype									
Aspirin + ticagrelor, % (*n*)	60.2 (284)	74.8 (459)	<0.001	59.9 (173)	70.7 (258)	0.002	60.7 (111)	80.7 (201)	<0.001
Aspirin + clopidogrel, % (*n*)	39.8 (188)	25.2 (155)	<0.001	40.1 (116)	29.3 (107)	0.002	39.3 (72)	19.3 (48)	<0.001
LVEF	55.1 ± 7.5	55.0 ± 7.5	0.830	54.6 ± 7.7	54.8 ± 7.7	0.792	55.8 ± 7.3	55.3 ± 7.2	0.451

AMI: acute myocardial infarction; NSTEMI: non-ST-segment elevation myocardial infarction; STEMI: ST-segment elevation myocardial infarction; DAPT: dual antiplatelet therapy; LVEF: left ventricular ejection fractions.

**Table 3:** Gender disparities about patients' decision and MACE in STEMI subgroup.

	Female (*n* = 183)	Male (*n* = 249)	*P*
Time from			
Chest pain to visiting hospital (*h*)	5.1 ± 5.8	4.6 ± 6.2	0.356
Diagnosis to consent to CAG (min)	34.1 ± 70.6	17.4 ± 24.0	0.015
Door-to-balloon (min)	82.4 ± 7.6	67.0 ± 6.0	0.031
CAG, % (*n*)	77.6 (142)	85.1 (212)	0.030
PCI/CABG, % (*n*)	64.5 (118)	75.1 (187)	0.011
Degree of adherence to			
DAPT	2.7 ± 1.2	3.0 ± 1.1	0.016
Statins	2.6 ± 1.4	3.1 ± 1.2	<0.001
Regular use of TCM, % (*n*)	55.7 (102)	45.8 (114)	0.026
MACE, % (*n*)	15.3 (28)	8.0 (20)	0.014

CAG: coronary angiography; MACE: major adverse cardiac event; NSTEMI: non-ST-segment elevation myocardial infarction; STEMI: ST-segment elevation myocardial infarction; PCI: percutaneous coronary intervention; CABG: coronary artery bypass grafting; DAPT: dual antiplatelet therapy; TCM: traditional Chinese medicine.

**Table 4:** Gender disparities about patients' decision and MACE in NSTEMI subgroup.

	Female (*n* = 289)	Male (*n* = 365)	*P*
Time from			
Chest pain to visiting hospital (h)	8.8 ± 8.1	6.6 ± 50	0.001
Diagnosis to consent to CAG (min)	75.6 ± 211.7	36.8 ± 87.2	0.005
Door-to-needle (h)	7.8 ± 7.5	6.5 ± 6.0	0.031
CAG, % (*n*)	73.4 (212)	81.1 (296)	0.012
PCI/CABG, % (*n*)	58.1 (168)	72.1 (263)	<0.001
Degree of adherence to			
DAPT	2.8 ± 1.2	3.1 ± 1.1	0.001
Statins	2.7 ± 1.3	3.1 ± 1.2	<0.001
Regular use of TCM, % (*n*)	47.1 (136)	36.2 (132)	0.003
MACE, % (*n*)	14.2 (41)	9.3 (34)	0.063

CAG: coronary angiography; MACE: major adverse cardiac event; NSTEMI: non-ST-segment elevation myocardial infarction; STEMI: ST-segment elevation myocardial infarction; PCI: percutaneous coronary intervention; CABG: coronary artery bypass grafting; DAPT: dual antiplatelet therapy; TCM: traditional Chinese medicine.

**Table 5:** Multivariate analysis about the risk factors for MACE.

	NSTEMI			STEMI		
	*P*	OR	95% CI	*P*	OR	95% CI
Gender	0.211	0.608	0.279–1.326	0.401	0.766	0.411–1.427
LVEF	0.001	0.926	0.886–0.968	<0.001	0.935	0.907–0.964
PCI/CABG	0.003	0.342	0.169–0.690	0.033	0.557	0.325–0.955
Adherence to DAPT	<0.001	0.554	0.424–0.726	<0.001	0.611	0.496–0.754
Adherence to statins	0.333	1.145	0.870–1.507	0.017	0.781	0.637–0.956
TCM	0.158	1.688	0.815–3.495	0.726	1.100	0.646–1.873

LVEF: left ventricular ejection fraction; CAG: coronary angiography; MACE: major adverse cardiac event; NSTEMI: non-ST-segment elevation myocardial infarction; STEMI: ST-segment elevation myocardial infarction; PCI: percutaneous coronary intervention; CABG: coronary artery bypass grafting; DAPT: dual antiplatelet therapy; TCM: traditional Chinese medicine.

## Data Availability

The data used to support the findings of this study are restricted by the Ethics Committee of the Fourth Affiliated Hospital Zhejiang University in order to protect privacy of all patients. The data are available from the corresponding author for researchers who meet the criteria for access to confidential data.

## Supplementary Materials

Supplementary Materials STROBE statement: checklist of items that should be included in reports of cohort studies.
